# Biomarkers of Oxidative Stress as Indicators of Fungi Environmental Pollution in Balb/c Albino Mice Monitored from South West, Nigeria

**DOI:** 10.1155/2019/6561520

**Published:** 2019-04-07

**Authors:** Adeyinka Odebode, Adedotun Adekunle

**Affiliations:** ^1^Department of Botany, Faculty of Science, University of Lagos, Akoka, Lagos, Nigeria; ^2^Department of Environment and Natural Science, Kabale University, Uganda

## Abstract

The presence and detection of common airborne fungi in an area are important for the prevention and treatment of allergic fungal diseases. Because of the ubiquitous nature of fungi, the effect of four different fungal species in production of antioxidant and reactive oxygen species production in balb/c albino mice was investigated. Fifty-four balb/c mice were randomly divided into eight groups (n = 6) and a normal control group. Four different fungal plates, comprising* Aspergillus flavus*,* Aspergillus penicillioides*,* Penicillium citrinum,* and* Penicillium chrysogenum,* which were the most abundant fungi species sampled in the environment were cultured for one week to make 2.3 x 10^7^ and 3.2 x 10^5^ spores and injected intranasally in sterile saline into the nostrils of each of the mice. Results showed that all fungal inoculated organism produced statistically (P<0.05) significant reactive oxygen species while antioxidant parameters were significantly decreased in a dose dependent manner compared with normal control mice. It is therefore concluded that* Aspergillus flavus*,* Aspergillus penicillioides*,* Penicillium citrinum,* and* Penicillium chrysogenum* can alter and decrease immune function in balb/c mice. Therefore, this study was conducted to identify the most common airborne fungal species present in Southwest Nigeria and to study their allergic reactions.

## 1. Introduction

The human airways are always continuously exposed to fungi which most times occur at higher number and for a longer duration than that of pollen or other known airborne allergens. The most common of these fungi are* Aspergillus *and* Penicillium* species. Sensitization to airborne fungal elements has been shown to be associated with asthma severity and later death [1]. Fungal air contamination has now become an environmental health issue because of the allergenicity of certain fungal spores which are present in both indoors and outdoors. Oxidative stress occurs not only as a result of inflammation but also from exposure to air pollution, cigarette smoke, and other factors such as inhalation of fungi spores and leads to the production of reactive oxygen species which is balanced by the action of antioxidants in the body [2]. Fungal spores and hyphae are present in the environment and respiratory exposure to them is almost constant throughout the year. The most common airborne fungi in the tropics include species found within the genera* Penicillium*,* Aspergillus*,* Fusarium*,* Cladosporium, *and* Alternaria*. Other sources of allergens include grass pollen, house dust mites, and cat dander but fungi alone have the ability to germinate and secrete additional molecules in the respiratory tract. Fungi can also colonize the respiratory tract. This is the greatest difference and probably the most important factor in the pathologic capabilities of fungi with regard to allergic inflammation. Thus, fungi have a much greater impact on an individual in terms of triggering host immune responses against potential pathogens from an allergenic angle while also producing nonallergenic toxins, enzymes, and other proinflammatory factors which may play a role in triggering and exacerbating chronic inflammatory disorders by stimulating innate immune responses and influencing the development of adaptive Th2 immunity (Babiceanu, 2011). Vulnerability to fungi occurs via ways such as inhalation, skin contact, or ingestion. The free radicals and reactive oxygen species are capable of damaging the integrity and also alter the function of biomembranes, which can lead to the development of many pathological processes (Faix et al. 2003). All organisms have evolved over time complex cellular defenses that are known as antioxidants to overcome toxicity caused by environmental factors. The disproportion between reactive oxygen species and antioxidants is called oxidative stress. Oxidative stress happens in many allergic and immunologic conditions. However, the primary defense against reactive oxygen species is endogenous antioxidants, which include the families of superoxide dismutase (SOD), catalase, glutathione peroxidase, glutathione S-transferase, and thioredoxin and low-molecular-weight compounds such as glutathione and lipoic acid [3]. Cytokines are released during the early phase of an immune response to allergen or foreign substance which triggers cellular inflammatory response over the next few hours which later results in recurrent symptoms in the host [4, 5]. Reactive oxygen species generation through bombardment of the system by intruders or other means is a constant problem for which cells have developed multiple protective mechanisms to survive. Bowler and Crapo, 2002 [6], reported that cigarette smoke inhalation results in increased exposure to both superoxide and hydrogen peroxide. Oxidative stress results when an imbalance occurs between the oxidative forces and the antioxidant defense systems in the body which is believed to favor an oxidative injury that has been implicated in the pathogenesis of asthma and other diseases (Marple, 2010). The role of oxidative stress as a result of fungi allergy has not been well studied. Assemblage of these antioxidants molecules differs depending of both subcellular and anatomic position. Many reports suggest that oxidative stress plays an important role in the pathogenesis of asthma and so many investigators have shown that increases in reactive oxygen species that occur during asthma are connected with destruction to a wide range of biological molecules in the lung. Not much work has been done in environmental sampling of airborne fungal spores present in the atmosphere in Nigeria and the consequences of inhaling the various fungi present in the air.

This work is therefore aimed to study the effect of four fungal species on antioxidant and reactive oxygen species production in lungs of balb/c mice.

## 2. Materials and Methods

### 2.1. Atmospheric Fungi Collection

Aero spores were sampled monthly for a period of two years. Open plate method was used for sampling by opening plates containing agar (Dichloran glycerol 18 and Potato Dextrose Agar) which was prepared under aseptic condition in the laboratory. Samples were collected in triplicate and transferred to the Mycology Laboratory of the Department of Botany, University of Lagos, and incubated at room temperatures (28-31°C) for 3 to 5 days. Colony count and growth appearance were monitored.

### 2.2. Identification of Fungi

Once there is presence of growth, the topography, texture, and pigmentation of each specific type of colony are noted in order to identify the fungi accurately. The identities of these fungi were identified using cultural and morphological characteristics as well as comparing them with confirmed representatives of different species in relevant texts such as Alexopolous et al. (2007), Barnett and Hunter (1999), and Ellis et al. (2007).

Molecular method was further employed in identification of sampled fungi due to limitations which exist in morphological identification (Table 1).

### 2.3. DNA Extraction

Extraction of fungal DNA was done using two different protocols, namely, modified cetyltrimethylammonium bromide (CTAB) protocol.

### 2.4. Gel Extraction and Sequencing

Gel extraction and sequencing were done at the Institute of Genomics core facility at the University of California, Riverside.

### 2.5. Primer and Amplicon Design

To make 100 *μ*M concentration stock solution of primers, the number of n moles in the tube is multiplied by 10 to give the amount of elution buffer to be used to dilute it after which 10 *μ*l was pipetted out and mixed with 90 *μ*l of elution buffer to give a working solution.

The target sequence selected was between 75 and 100 bp long with a GC content between 50 and 60% which did not contain secondary structures. The primers had a melting temperature of between 55 and 65°C. Oligonucleotides were designed using Primer-Blast, a program developed by NCBI that uses the algorithm Primer 3. Primer sequences were compared (blasted) to the user-selected databases to ensure they are unique and specific for the gene of interest.

The experiment was in accordance with animal welfare. Eighty balb/c mice (5-6 weeks old) were purchased from Veterinary Teaching Hospital of the University of Ibadan and kept in the animal house of Biochemistry Department. The animals were acclimatized for one week prior to experimental set-up. They were fed mice pellet purchased from Ladokun factory, Ibadan, and water* ad libitum. *After acclimatization, mice were put six in each cage. Four fungi species,* Aspergillus penicillioides*,* A. flavus*,* Penicillium citrinum,* and* P. chrysogenum,* were isolated and subcultured till a pure culture was obtained and made into two different concentrations ((2.3 x 10^7^ and 3.2 x 10^5^) before intranasal instillation in mice, thus, a total of eight groups and one control group which was without any fungal inoculation.

### 2.6. Fungi Inoculation

*A. flavus, A. penicillioides, Penicillium chrysogenum, and P. citrinum *were isolated from various environments in Lagos and Ibadan, Nigeria, and grown to stationary phase (72 h) at 37°C on potato dextrose agar (PDA). The conidia were harvested by gentle washing with sterile endotoxin-free phosphate buffered saline (PBS). The resulting fungal suspension was then filtered through two layers of sterile gauze to remove hyphae. The cultures were then washed in aseptic nonpyrogenic saline and counted using a hemocytometer. Mice were inoculated with the different fungi at two different concentrations (2.3 x 10^7^ and 3.2 x 10^5^) by intranasal administration using a 1 ml syringe in each nostril.

Before the animals were inoculated intranasally, mice were anesthetized by intraperitoneal injection with a ketamine-xylazine solution (2.5 mg of ketamine (Fort Dodge Animal Health, Fort Dodge, Iowa).

### 2.7. Dissection for Analysis

The lungs were aseptically removed and placed in 10 ml of 10% formalin. The lungs (filled with 10% phosphate buffered formalin) were preserved in 10% phosphate buffered saline for histopathological evaluation.

### 2.8. Evaluation of Biomarkers of Oxidative Stress

Animal tissues (lungs) were rinsed with 1.15% KCl/4_C solution. After that, 60% tissue homogenates were prepared with 0.1M phosphate buffer (pH 7.4/37°C). Supernatants for biochemical assays were prepared after separation of the nuclei and mitochondrial fractions at 10,000 g/4°C 12 min from 60% tissue homogenate. Reduced glutathione (GSH) was determined according to Jollow et al. Lipid peroxidation was determined as malondialdehyde (MDA) according to the procedures described by Varshney and Kale. The nitrite (NO2 –) level in the tissues was estimated as an index of nitric oxide (NO) production. Quantitation was based on the Griess reaction as described by Crespo et al. Myeloperoxidase (MPO) activity, an indicator of polymorphonuclear leukocyte accumulation and activation, was determined by the method describe by Bradley et al. The protein concentrations of the various samples were determined by means of the Biuret method as described by Gornal et al. (1949), a slight modification: potassium iodide was added to the reagent to prevent precipitation of Cu^2+^ ions as cuprous oxide. The level of SOD activity was determined by the method of Misra and Fridovich (1972).

The method of Beutler et al. (1963) was followed in estimating the level of reduced glutathione (GSH).

Hydrogen peroxide generation was determined according to the method of Galli F. et al., 2005.

### 2.9. Statistical Analysis

Data obtained were analysed using multiple analysis of variance (ANOVA) and means were separated using Duncan Multiple Range Test (DMTR) with the level of significance at P<0.05 (95% confidence interval).

## 3. Result

The fitted model of the analysis of variance of effect of fungal spores at different inoculum load of fungi spores on biochemical parameters in lungs of balb/c mice produced a highly significant (p<0.01) effect on all reactive oxygen species and antioxidant parameters except for hydrogen peroxide which showed no significant difference for both treatments and inoculum concentration. A significant (p<0.05) effect was also observed for inoculum load on myeloperoxidase (MPO), superoxide dismutase (SOD), and glutathione (GSH) (Table 2).

The fitted model of the analysis of reactive oxygen species and antioxidant parameters on lungs of balb/c mice inoculated with fungi spores showed that control was significantly higher for nitric oxide (NO) while lower in malondialdehyde (MDA), myeloperoxidase (MPO), and hydrogen peroxide (H_2_O_2_). Result for antioxidant parameters show that control was higher in glutathione

(GSH), protein content, and superoxide dismutase (SOD). There was no significant difference in the inoculum load for the two concentrations except for MPO (reactive oxygen species) while SOD and GSH for (antioxidant parameters) also showed significant difference between the inoculum loads (Table 3).

Negative and significant (p≤ 0.05) correlation exist between fungal treatments and protein (- 0.55), SOD (-0.71), nitric oxide (-0.44), and GSH (-0.56) while positive and significant ((p≤ 0.05) correlation exist between fungal treatments hydrogen peroxide and MPO. Variations in the inoculum load produced negative but not significant (p≤ 0.05) correlation with protein, SOD, nitric oxide, and GSH but significant (p≤ 0.05) effect exists with MPO (r = 0.31). Replicated treatments produced positive but not significant (p≤ 0.05) effect on MDA, hydrogen peroxide, nitric oxide, and GSH (Table 4).

**Figure 1:**
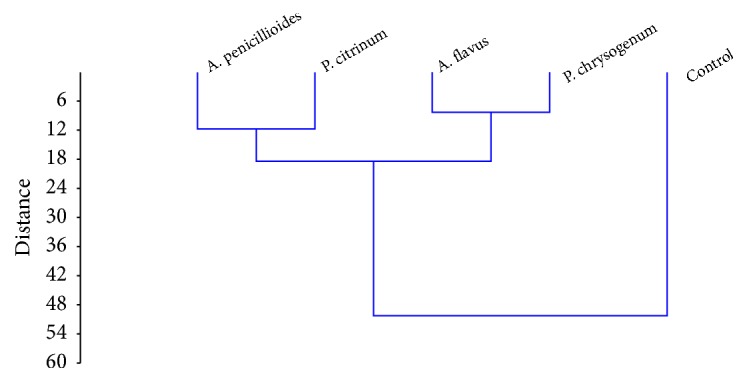
Cluster diagram showing the relationship in the biochemical performances of the fungi.

The cluster diagram showing the relationship in the different fungi inoculated in mice showed that there were two main clusters with control standing alone and all the four inoculated also clustered in two distinct clusters.* Aspergillus penicillioides *and* Penicillium citrinum *were on the same subcluster while* Aspergillus flavus *and* Penicillium chrysogenum *also subclustered together. Control was alone on a separate cluster (Figure 1). Contribution of Principal component analysis showed similar level of distribution of the principal components within the antioxidants and reactive oxygen species. Prin 1 with the highest Eigen proportion was found mostly related to SOD and nitric oxide. Prin 2 with Eigen proportion of 24.74 was found to majorly contribute to the increase in MDA and protein. The fourth principal component showed strong increase with hydrogen peroxide while the sixth component showed very strong correlation with SOD (Table 5).

**Figure 2:**
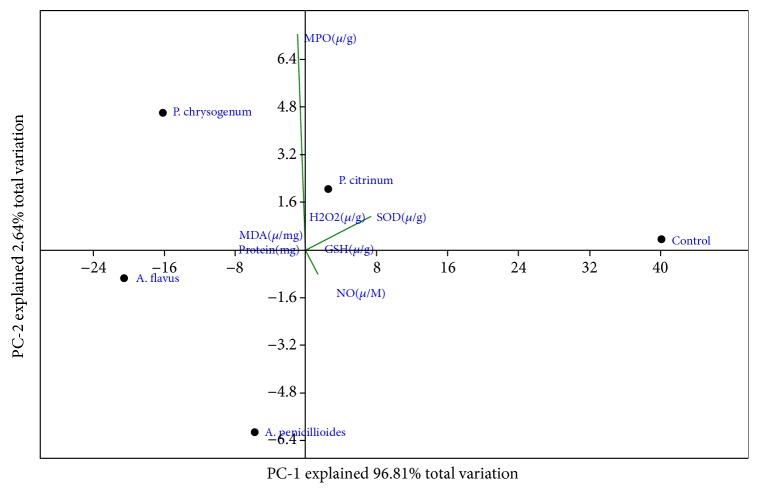
Contribution of PC 1 and PC 2 to the variation in the biochemical properties of the lungs caused by the fungi spores.

The scatter plot showed variations of the Principal Component Axis in the activities of the inoculated organisms on the biochemical parameters of mice lungs. The PC1 which explained 96.81 % of the total variations recorded positive association with SOD, GSH, and control with* P. chrysogenum*,* P. citrinum,* and* A. penicillioides* while nitric oxide and protein content with* A. flavus* showed negative relationship with Prin 1 with nitric oxide expressing stronger association. The PC 2 explained 2.64% of the total variation and showed positive association with control and SOD experiment while all other biochemical parameters were negatively associated with PC 2 (Figure 2).

**Figure 3:**
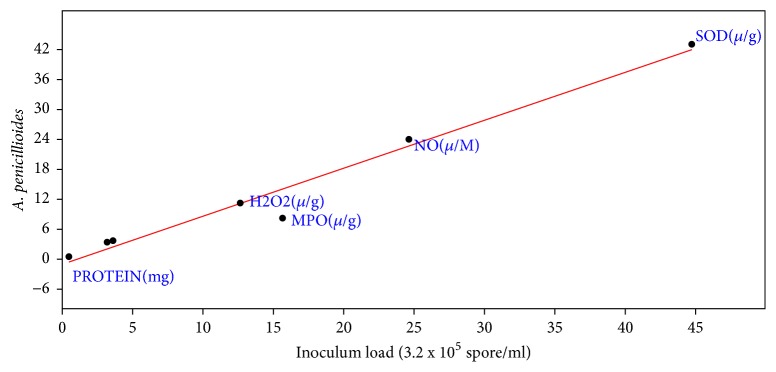
Correlation coefficient the effect of inoculums load (3.2 x 10^5^ spores/ml) of* A. penicillioides* on the biochemical properties of the lung (r= 0.99, slope = 0.96+0.07, intercept = -0.94+1.54, and p = 4.77 x 10-^5^).

**Figure 4:**
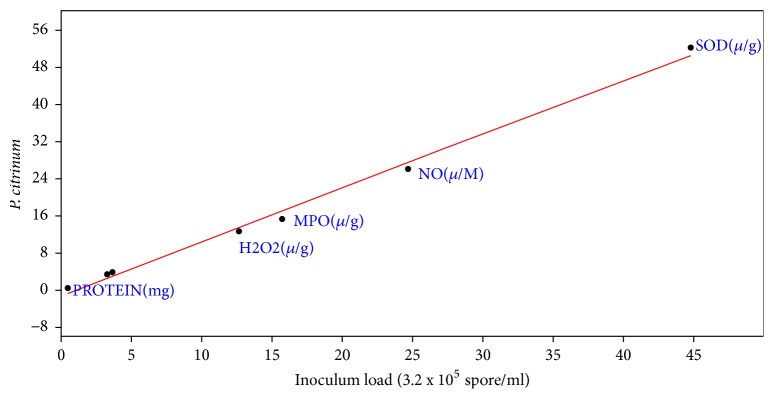
Correlation coefficient the effect of inoculums load (3.2 x 10^5^ spores/ml) of* P. citrinum* on the biochemical properties of the lung (r= 0.997, slope = 1.16+0.04, intercept = -1.03+0.77, and p = 6.14 x 10-^7^).

**Figure 5:**
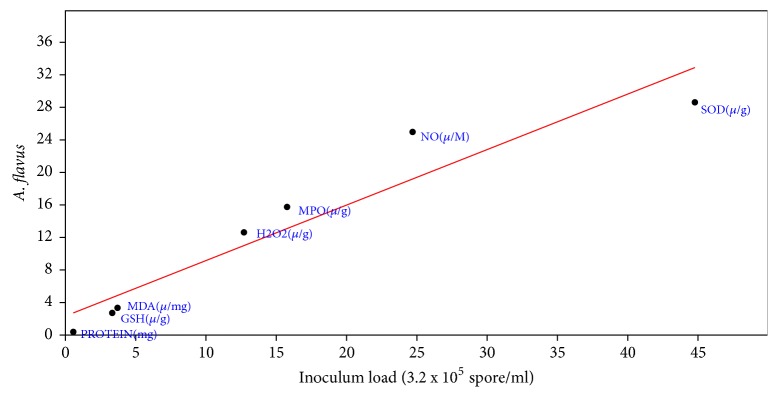
Correlation coefficient the effect of inoculums load (3.2 x 10^5^ spores/ml) of* A. flavus* on the biochemical properties of the lung (r= 0.95, slope = 0.68+0.10, intercept = 2.42+2.07, and p = 0.001).

**Figure 6:**
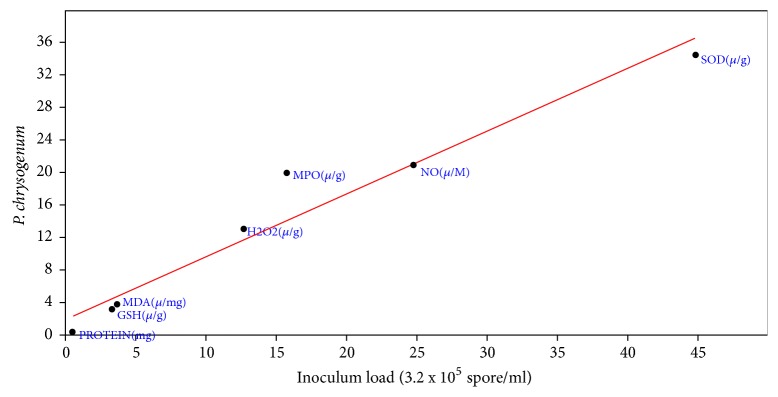
Correlation coefficient the effect of inoculums load (3.2 x 10^5^ spores/ml) of* P. chrysogenum* on the biochemical properties of the lung (r= 0.97, slope = 0.77+0.08, Intercept = 2.22+1.68, and p = 0.00022).

Correlation coefficient the effect of inoculums load of* A. penicillioides* on the biochemical properties of inoculated mice showed that r was 0.99, slope was 0.96+0.07, intercept was -0.94+1.54, p = 4.77 x 10-^5^ (Figure 3). For* P. citrinum* inoculated mice, correlation coefficient of effect of inoculums load showed r to be 0.997, slope = 1.16+0.04, intercept = -1.03+0.77, and p = 6.14 x 10-^7^ (Figure 4). Correlation coefficient of inoculums load of* A. flavus* on the biochemical properties of the inoculated mice also showed r= 0.95, slope = 0.68+0.10, Intercept = 2.42+2.07 and P to be 0.001 (Figure 5). For* P. chrysogenum *inoculated mice, r was 0.97, slope = 0.77+0.08, intercept = 2.22+1.68, and p = 0.00022 (Figure 6).

## 4. Discussion

The role of fungi in allergic diseases is well documented in literature. Airborne pollen and fungal allergenic spores have been implicated as one of the main cause of allergic respiratory diseases in temperate regions [7] but less is known about their allergenicity in the tropics. The dominant species of airborne fungi in South West Nigeria atmosphere monitored throughout the year sampled were* Aspergillus *spp. and* Penicillium* spp. which necessitated their use in intranasal instillation in mice. Fungal disease cause a wide range of diseases that include allergies, superficial infections, and invasive mycoses [8], which are often associated with high rates of morbidity and mortality [9]. It is generally accepted that antioxidants are important reactive oxygen species counterbalance and defend the organism from exceeding oxidative stress. Reactive Oxygen Species (ROS) have been shown to be a building block of the killing response of immune cells to microbial disruption. Current affirmation has shown that ROS play a key role as an agent in normal cell signal transduction and cell cycling. Hancock et al., 2001 [10], in their work demonstrated that ROS have a role in cell signaling, including; apoptosis; gene expression; and the activation of cell signaling cascades. Cellular protection against ROS removal of reactive oxygen species is prime to the survival of all aerobic life forms. As a result of this, a number of barricade mechanisms have evolved to meet this need and provide a balance between production and removal of ROS. Cells have categories of fortifying mechanisms to ameliorate the harmful effects of ROS. Superoxide dismutase (SOD) catalyzes the conversion of two superoxide anions into a molecule of hydrogen peroxide (H_2_O_2_) and oxygen (O_2_) while glutathione peroxidase is a group of enzymes containing selenium, which also speed up the wearing down of hydrogen peroxide, as well as organic peroxides to alcohols. Glutathione is the most important nonenzymatic oxidant barricade mechanism. It occurs in relatively large amounts (mM levels) and available to detoxify peroxides and restore a number of important antioxidants.

Hydrogen peroxide (H_2_O_2_) is the most important ROS with regard to cell cycle regulation. Nitric oxide has many physiologic functions and physiopathological effects in the organism and is synthesized by the enzymes iNOS/nNOS/eNOS from L-arginine. Nitric oxide is accepted as an antioxidant by capturing radical O- and as an oxidant by forming peroxynitrite [11].

In the present study, both malondialdehyde (MDA) and myeloperoxidase (MPO) concentration in the lung tissue increased significantly.* Penicillium citrinum* had the highest effect on MDA while* Aspergillus flavus* inoculated fungi had least amount of MDA measured. For MPO,* P. chrysogenum* inoculated mice had highest level measured in the lungs. Hirvonen et al., 1997c [12], similarly observed that the spores of* S. californicus *induced the production of reactive oxygen species (ROS) in both human polymorphonuclear leucocytes and mouse macrophages* in vitro.*

In our result, NO and MDA were not significant for fungal concentration because the concentration of the inoculum may have not been sufficient enough to cause any infection in the mice. H_2_O_2_ was also not significant because it is an inflammation marker which correlates with severity of diseases and hence its presence may not be sufficient enough to cause significant damage in the system going by the inoculum load of the fungal species. Another explanation may be that the enzymatic defense activity of GSH has prevented the formation of hydroxyl radical (OH).

Protein abundance as revealed in this study did not also vary among the four inoculated fungi likewise the different concentrations also did not affect its production in lungs. For MDA,* P. chrysogenum* and* P. citrinum* expressed more of the reactive oxygen species compared to* A. penicillioides* and* A. flavus* while the inoculum load also did not statistically affect elevation level in lungs.* P. chrysogenum* had the highest elevation of lung MPO while the least expressed was found in* A. penicillioides* inoculated mice. The inoculum load showed variation in expression level which confirmed the effect of load dose on the expression of reactive oxygen species. Hydrogen peroxide level was elevated in* P. chrysogenum* inoculated mice and lowest abundance was found in* A. penicillioides* inoculated lungs. The abundance did not vary with concentration load. The production of SOD showed its counter effect on the activities of the reactive oxygen species.* P. citrinum* inoculated mice produced more SOD than* P. chrysogenum*,* A. flavus,* and* A. penicillioides*. The higher the inoculum load the more SOD produced was also observed. GSH abundance was more in* P. citrinum* inoculated mice and lowest abundance was measured in* A. flavus* inoculated tissue. The different concentration did not affect its abundance. The nitric oxide produced was the highest in* P. citrinum* inoculated mice while the lowest measurement was observed in* P. chrysogenum*. Inoculum load did not also show relationship with its abundance. It has been shown recently that human macrophages produce NO in several inflammatory conditions, including tuberculosis, rheumatoid arthritis, and malaria [3]. NO take part in antimicrobial host defense against extracellular pathogens in human as well as in murine macrophages [3, 13]. Glutathione is known to react directly with ROS and acts as a nonenzymatic antioxidant [14]. This study revealed that glutathione was more expressed by* P. citrinum *inoculated mice. A similar response to fungi spore challenge in C57BL/6 mice has been reported previously by Noverr et al., 2004 [15], who observed disrupted microbiota and immune response in the airways to fungi exposure.

Also in our study, the dendrogram showed the four fungal species cluster differently because* A. penicillioides* and* P. citrinum* have similar effect on the lungs of the mice while* A. flavus* and* P. chrysogenum* activities also had similar effect on the lungs.* A. flavus* and* P. chrysogenum* activities on the lungs caused higher production of MDA in the cells (more ROS) produced. Their activity in the system is lethal compared with spores of* P. citrinum* and* A. penicillioides*. The activities of these four fungal species differ in the production of ROS which has been shown in this work.

The present investigation therefore examined changes in several measures of oxidative stress and antioxidant status in fungi allergy. Taken together, these results provide new information on the role of fungi on oxidative stress and antioxidant level in mice.

## 5. Conclusion

The observations that the inoculated microbes cause inflammation and toxic responses in the lungs suggest that they can contribute to inflammation associated adverse health effects such as allergy which result in oxidative stress damage. When compared based on comparable volumetric doses, the fungal species* P. chrysogenum *and* P. citrinum *were more potent at inducing adverse effects than the fungal species* A. penicillioides *and* A. flavus*.

## Figures and Tables

**Table 1:** Primers used for fungi amplification.

Locus	Primer name	Direction	Sequence	Target region
Internal Transcribed Spacer (ITS)	ITS 1	Forward	5′TCCGTAGGTGAACCTGCGG3′	18S rDNA
	ITS 4	Reverse	5′TCCTCCGCTTATTGATATGC3′	
Large Ribosomal Unit	LRO5	Forward	5′TCCTGAGGGAAACTTCG3′	LSU
	LROR	Reverse	5′ACCCGCTGAACTTAAGC 3′	

**Table 2:** ANOVA effect of fungal treatments at different inoculum load on the biochemical properties of lungs of balb/c albino mice.

Source	df	Protein	MDA	MPO	H_2_O_2_	SOD	NO (*µ*/M)	GSH
Model	9	0.003^*∗*^	0.313^*∗∗*^	135.7^*∗∗*^	3.54	2585.05^*∗∗*^	127.40^*∗*^	1.378^*∗∗*^
Treatments	4	0.007^*∗∗*^	0.64^*∗∗*^	246.78^*∗∗*^	6.06	5533.38^*∗∗*^	234.67^*∗*^	2.43^*∗∗*^
Concentration	1	0.0009	0.05	197.00^*∗*^	5.04	1072.55^*∗*^	96.07	1.00^*∗*^
Replicate	4	0.0003	0.04	9.29	0.65	14.85	27.95	0.417^*∗*^
Error	40	0.05	3.25	812	108.15	149.01	2152.56	5.87
Corrected total	49	0.085	6.07	2033.3	140.08	29226.3	3299.2	18.277

*Note.* ^*∗∗*^  = highly significant (p<0.01), ^*∗*^  = significant (p<0.05), and ns = not significant.

MDA: malondialdehyde, MPO: myeloperoxidase, *H*_*2*_*O*_*2*_: hydrogen peroxide, SOD: sodium dismutase, and NO: nitric oxide.

**Table 3:** Effect of inoculated fungi on the biochemical properties of lungs of balb/c albino mice.

Parameters	Variables	Protein (mg)	MDA (*µ*/mg)	MPO (*µ*/g)	H_2_O_2_ (*µ*/g)	SOD(*µ*/g)	NO (*µ*/M)	GSH (*µ*/g)
Treatments	Control	0.516a	3.384b	8.977c	11.65ab	88.36a	34.00a	4.08a
	*A. penicillioides*	0.480b	3.573b	8.334c	11.35b	43.08bc	24.03b	3.41bc
	*P. citrinum*	0.457b	3.868a	15.321b	12.69ab	52.15b	26.13b	3.59b
	*A. flavus*	0.461b	3.320b	15.795b	12.71ab	28.72d	25.00b	2.74d
	*P. chrysogenum*	0.445b	3.846a	20.071a	13.18a	34.55cd	21.00b	3.23c
	LSD	0.033	0.28	4.07	1.49	11.03	6.63	0.35
Inoculum load (spores/ml)	2.3 x 10^7^	0.48a	3.56a	11.72b	12.00a	54.00a	27.42a	3.55a
	3.2 x 10^5^	0.47a	3.63a	15.68a	12.63a	44.74b	24.64a	3.27b
	LSD	0.02	0.16	2.58	0.94	6.98	4.19	0.22
Replicate	1	0.47a	3.60a	14.54a	11.92a	49.88a	27.28a	3.35b
	2	0.46a	3.60a	13.68a	12.30a	49.80a	25.38a	3.19b
	3	0.47a	3.52a	12.58a	12.58a	50.13a	24.07a	3.74a
	4	0.47a	3.70a	14.77a	12.52a	47.20a	25.24a	3.35b
	5	0.46a	3.56a	12.91a	12.29a	49.84a	28.20a	3.45ab
	LSD	0.03	0.26	4.07	1.49	11.03	6.63	0.35
	EMS	0.001	0.08	20.3	2.7	149.02	53.81	0.15

Means with different letter across the column are significantly (p<0.05) different from one another with respect to each parameter.

**Table 4:** Association between the treatments, inoculums load, and biochemical properties of the lungs of balb/c albino mice.

Correlation	Fungi	Inoculum load	Replicate	Protein	MDA	MPO	H_2_O_2_	SOD	NO (*µ*/M)	GSH (*µ*/g)
Fungi	1									
Inoculum load	0	1								
Replicate	0	0	1							
Protein	-0.55^*∗*^	-0.11	-0.03	1						
MDA	0.27	0.09	0.01	-0.65^*∗∗*^	1					
MPO	0.66^*∗∗*^	0.31	-0.05	-0.55^*∗*^	0.34	1				
H_2_O_2_	0.38	0.19	0.08	-0.48	0.25	0.47	1			
SOD	-0.71^*∗∗*^	-0.19	-0.02	0.46	-0.12	-0.34	-0.21	1		
NO (*µ*/M)	-0.44	-0.17	0.03	0.46	-0.23	-0.19	-0.03	0.62^*∗∗*^	1	
GSH	-0.56^*∗*^	-0.24	0.09	0.38	-0.04	-0.48	-0.21	0.65^*∗∗*^	0.31	1

^*∗*^ Correlation is significant at p<0.05 and ^*∗∗*^ correlation is highly significant at p<0.01.

**Table 5:** Contributions of principal component axis (PCA) to the distribution of biochemical parameters.

PCA	PC 1	PC 2	PC 3	PC 4
Protein (mg)	0.001	-0.003	0.002	-0.046
MDA (*µ*/mg)	-0.003	0.032	-0.097	0.784
MPO (*µ*/g)	-0.129	0.969	0.127	-0.085
H_2_O_2_ (*µ*/g)	-0.018	0.157	0.066	0.439
SOD (*µ*/g)	0.974	0.152	-0.165	-0.027
NO (*µ*/M)	0.186	-0.108	0.967	0.095
GSH (*µ*/g)	0.019	0.007	-0.093	0.416
Eigen value	583.205	15.906	3.321	0.021
Percentage variance	96.805	2.640	0.551	0.003

## Data Availability

The data used to support the findings of this study are available from the corresponding author upon request.
